# Melanisation in Salmonid Skeletal Muscle: A Review

**DOI:** 10.1111/jfd.14063

**Published:** 2024-12-11

**Authors:** Håvard Bjørgen, Espen Rimstad, Erling Olaf Koppang

**Affiliations:** ^1^ Unit of Anatomy, Faculty of Veterinary Medicine Norwegian University of Life Sciences Ås Norway; ^2^ Unit of Virology, Faculty of Veterinary Medicine Norwegian University of Life Sciences Ås Norway

**Keywords:** chronic inflammation, fat necrosis, granuloma, inflammation, melanin, tyrosinase

## Abstract

Melanisation can occur in the musculature of fish. A well‐known form is the melanised focal changes, or ‘black spots’, in the fillet of farmed Atlantic salmon ( *Salmo salar* ). The aetiology of black spots has not been fully determined, though recent research has emphasised the role of fat necrosis in their development. The initial stages of the changes are observed as focal haemorrhages or ‘red spots’, and these can progress into melanised focal changes (MFCs). The focal haemorrhages are acute changes characterised by necrotic myocytes and adipocytes and diffuse haemorrhage in the tissue. These changes evolve into a chronic inflammation dominated by fibrosis, encapsulated lipid droplets or pseudocysts, presence of epithelioid cells, granulomas of varying character, giant cells and melano‐macrophages, whose presence accounts for the discolouration. The inflammation ranges from mild to severe, and the severity of the lesion has been associated with localised piscine orthoreovirus 1 (PRV 1) replication in macrophages and melano‐macrophages within granulomas. The possibility of a genetic impact on the condition has not been supported by available data. The lipid composition and the antioxidative properties of the feed have been shown to affect the development of the changes. Physiological and environmental factors are also believed to influence the prevalence and severity of the condition. Here, we review the current state of knowledge concerning melanisation in fish skeletal musculature, with a special emphasis on the MFCs in Atlantic salmon.

## 1 Introduction

In 2005, the first description of a phenomenon commonly known as ‘black spots’ in the musculature of Atlantic salmon ( *Salmo salar* ) was published (Koppang et al. 2005). These black spots were later referred to as melanised focal changes (MFCs) (Bjørgen et al. 2015)—an acronym that has been predominantly used in scientific literature since. The initial publication from 2005 marked the beginning of research efforts aimed at resolving the condition, which has been a significant problem for the fish farming industry. Histologically, the MFCs are characterised by chronic inflammation with the presence of epithelioid cells, multinucleated giant cells and extracellular lipid accumulations. In addition, the involvement of melanised cells accounted for the observed black discolouration of the changes. These cells have traditionally been termed ‘melano‐macrophages’ and are primarily observed in the teleost fish kidney and spleen but may also be seen in other visceral tissues (Bjørgen and Koppang 2021). Evidence suggests that melano‐macrophages constitute a leukocyte population capable of melanin synthesis found in ectothermic vertebrates (Bjørgen and Koppang 2024).

Melano‐macrophages typically occur at sites of inflammation, and their presence constitutes the signature cell component in inflammation‐related MFCs in the white muscle of Atlantic salmon (Bjørgen and Koppang 2024). Intraperitoneally administered vaccines in fish may trigger severe granulomatous inflammation with melano‐macrophages in the abdominal cavity (Poppe and Breck 1997), which is a phenomenon also observed in the musculature when oil‐based vaccines are deposited there (Mo et al. 2023). However, inflammation in fish can occur without the presence of such cells, for example, they appear not to participate in intestinal chronic inflammation in salmonids (Bjørgen, Li, et al. 2020). Despite the presence of melano‐macrophages in various immune organs and inflammatory conditions, numerous unresolved and fundamental questions about them remain (Bjørgen and Koppang 2024), frequently excluding them from being mentioned in comprehensive reviews addressing teleost immune cells.

Bjørgen et al. (2015) showed that MFCs represent the late stage of a chronic inflammatory condition, initially appearing as focal haemorrhages visible as red spots in the fillet. These haemorrhages, termed red focal changes (RFCs), were histologically characterised by extravascular erythrocytes, necrosis and acute inflammation. While RFCs themselves have been considered a minor issue at slaughter due to their relatively low prevalence and are less visible compared to MFCs, the latter affect approximately 20% of all salmon produced in Norwegian fish farms, leading to significant economic losses through fillet downgrading (Mørkøre et al. 2015). The situation has not improved since this report. For a long time, the underlying causes of the condition remained elusive, making it best characterised as a focal idiopathic inflammatory myopathy. However, recent research has provided valuable insights into the mechanisms driving these changes (Bjørgen, Brimsholm, Asserson et al. 2024), instilling hope for significant advancements in management strategies to reduce their prevalence.

In this review, we will not exclusively refer to peer‐reviewed published information concerning this topic. Some information has only been made available in the form of reports issued by the host institutions. Although these reports are primarily written in Norwegian, English abstracts are provided. Given the nature of these reports, the description of materials, methods and results may not be as well‐documented and as comprehensive as in a peer‐reviewed article. Nevertheless, this information may still be valuable. We will, therefore, present selected information from several reports (Mørkøre et al. 2015 Report 35/2015, Mørkøre et al. 2016 Report 31/2016; Bjørgen et al. 2018 Final technical report FHF project 901221; Lund et al. 2018 Report 22018, Mørkøre et al. 2022 Report 27/2022). A limited number of master's theses are also referred to. These sources will be referenced when we assess the information they provide unavailable elsewhere. Additionally, we will present a limited number of undocumented observations. These will be clearly indicated as such within the text, and readers should interpret this information with caution.

Scientific literature on MFCs originates primarily from the Norwegian salmon industry, although discussions with industry representatives from other salmon‐producing countries have indicated that similar problems exist there as well. However, comprehensive investigations in these countries are lacking. Consequently, our review is limited to the Norwegian context, but the factors discussed herein may also apply to other salmon‐producing countries. In sum, our aim is to offer a comprehensive review of the current understanding of melanisation in the fish musculature.

## 2 Muscle of Fish

Before we address MFC's themselves, a brief overview of fish musculature anatomy is appropriate. Despite the focus on muscle tissue, or fillet, in both farmed and wild‐caught fish within aquaculture production, relatively little attention has been paid to musculature‐related diseases in fish. In anatomy textbooks, muscle tissues are divided into smooth musculature, cardiac musculature and skeletal musculature. The latter may be further divided into red and white muscles. The skeletal musculature is primarily regarded as a tissue for movement, with limited consideration for other functions. However, recent research has highlighted the immunological benefits of muscle activity, with myocytes serving as significant sources of glutamine and interleukin‐6 (IL‐6) production (Rogeri et al. 2020). Under normal conditions, the musculature contains scattered macrophages that can be activated if required and be a source of inflammatory mediators (Rogeri et al. 2020; Tidball and Villalta 2010). Importantly, the musculature of salmonid fish species also constitutes a significant portion of fat‐containing cells, that is, adipocytes. In other species, adipocytes are increasingly being studied in the context of their whole‐body immunometabolic influence (Trim and Lynch 2022).

Regarding striated skeletal muscle, the organisation in fish is unique. Skeletal muscle is arranged in myotomes shaped like lying ‘W's, separated by myosepta defined as fibro‐collagenous partitions that physically segregate myotomes and provide attachment points for muscle cells. The myosepta of salmonids contain a substantial portion of adipose tissue, but also vessels and nerves. The aerobic red musculature is located beneath the skin and centred around the ventral mid‐line, while the anaerobic white muscle is situated beneath and constitutes the bulk of the skeletal musculature and thus unlike the situation in birds and mammals being arranged in separate musculature compartments (Kryvi and Poppe 2016; Limbach et al. 2022; Zhol, Ackman, and Morrison 1995).

To understand the potential basis for pathological changes in the muscle of salmonids compared with mammals, it is important to highlight certain differences. In the Atlantic salmon, the musculature is especially rich in adipocytes (Nanton et al. 2007; Zhol, Ackman, and Morrison 1995). Their abundant presence in the musculature may therefore be of particular importance when validating musculature changes in such species. In Atlantic salmon, adipocytes within the myosepta serve as the primary site of lipid storage. In older fish, fatty infiltrations into the myotomes can also be seen. Additionally, intracellular lipid storage has been observed in red myocytes but not in white myocytes (Nanton et al. 2007; Zhol, Ackman, and Morrison 1995). Regarding the gross anatomical distribution of fat, Katikou, Hughes, and Robb (2001) categorised the fillet musculature into nine zones and noted that the abdominal musculature exhibited particularly high lipid content. Conversely, the dorsal musculature showed lower levels, while the leanest portions were observed in the caudal musculature. Another study, employing a slightly different fillet division, found relatively high levels in the abdominal musculature compared to other locations. Also, a consistent fatty acid composition across the various locations was confirmed (Refsgaard, Brockhoff, and Jensen 1998). As will be later discussed, this information holds significant relevance when evaluating muscle changes at different fillet locations.

Another important trait of salmonid musculature is its ability to store astaxanthin, accounting for the red colour of the tissue. Additionally, astaxanthin serves as a potent antioxidant. Refsgaard, Brockhoff, and Jensen (1998) observed the highest concentration of astaxanthin in the caudal and dorsal regions of the fillet musculature compared with the anterior portions, which also contain higher lipid content. Grünenwald et al. (2020) suggested that differences in the astaxanthin binding capacity of myofibrillar proteins at different anatomical muscle locations may account for the observed variation.

To summarise the most important differences between salmonid and mammalian musculature, the compartmentalisation of red versus white musculature, the abundant storage of lipids and finally the content of astaxanthin appear as the most striking features of salmonid musculature.

## 3 Melanin Synthesis and Properties of Melanin

**FIGURE 1:**
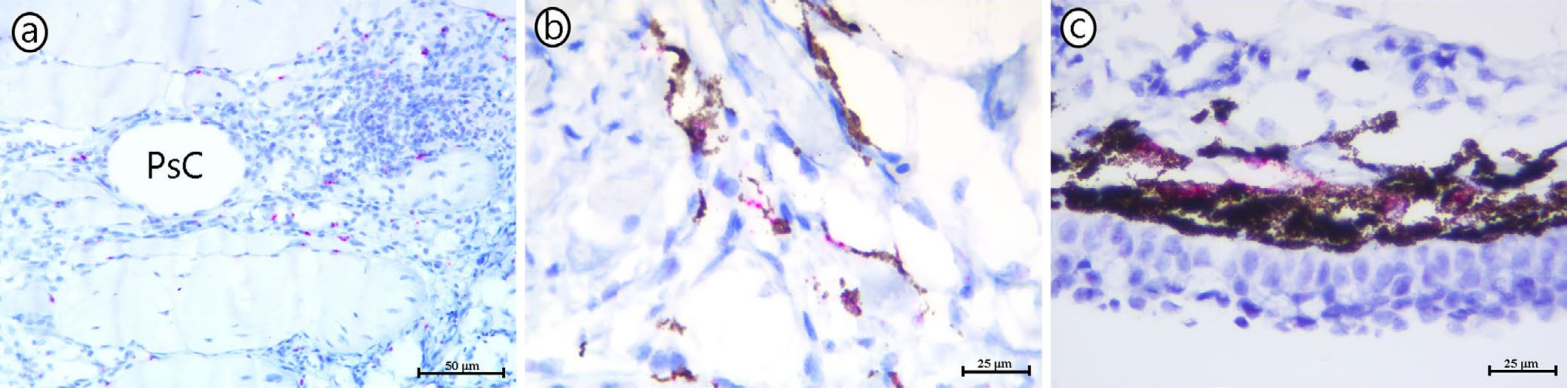
In situ hybridisation targeting tyrosinase. (a) Multiple tyrosinase‐positive (red colour) amelanotic cells in an inflammatory cell infiltrate in a red focal change. PsC—pseudocyst. Scale bar—50 μm. (b) Tyrosinase‐positive (red colour) pigmented cells within a melanised focal change. Scale bar—25 μm. (c) Tyrosinase‐positive (red colour) melanophores in the dermal layer of the skin. Scale bar—25 μm.

Melanin is a complex compound that can be found in different forms throughout nature. Black eumelanin, reddish pheomelanin and neuromelanin have been isolated in higher vertebrates (Cao et al. 2021), but in fishes, only eumelanin is thought to be produced (Kottler, Künstner, and Schartl 2015). Eumelanin is formed in intracellular organelles called melanosomes through the actions of enzymes of the tyrosinase gene family (Figure 1) comprising tyrosinase, tyrosinase‐related protein‐1 (Tyrp1) and tyrosinase‐related protein‐2 or dopachrome tautomerase (Tyrp2/Dct), and this system has been identified and studied in fish including melanised focal changes (Bjørgen, Brimsholm, Asserson, et al. 2024). Eumelanin was, therefore, not surprisingly identified in these changes (Wakamatsu et al. 2023). It is noteworthy, however, that even if the enzymes at work appear to be the same between fish and mammals, the genesis of melanosomes of visceral organ‐derived melanin‐producing cells in fish is different. Here, sheets of membranes split off the Golgi apparatus form the spherical melanosomes (Haugarvoll et al. 2006) in a similar fashion as that observed in amphibia Kupffer cells (Sichel et al. 1997). Thus, the intralumenal Pmel17 fibrils essential for melanosome formation in mammals (Raposo and Marks 2007) are not involved. This could have a fundamental, yet undiscovered implication for immune functions as the genesis of mammalian melanosomes is closely related to Mhc class II^+^ compartments (Raposo and Marks 2007), whereas this does not seem to be the case with fish melanosomes.

When present in the skin, melanin has an obvious function as a compound protecting the organism from solar radiation, camouflaging from predators and for signalling to other individuals (Nüsslein‐Volhard and Singh 2017). As we recently discussed in a previous review with special reference to melano‐macrophages in fish, the function of extracutaneous melanin is less obvious (Bjørgen and Koppang 2024). There is an astonishing dualism between the properties of melanin (d'Ischia et al. 2015) and melanogenesis. On one hand, during melanogenesis, free radicals are formed that may be harmful not only to the melanocytes themselves but also to the surrounding tissue, and therefore, several protective mechanisms are present (Land, Ramsden, and Riley 2006; d'Ischia et al. 2015). In invertebrates, this reaction is used to inactivate pathogens and possibly also isolate them in newly synthesised melanin. We have speculated if similar functions can be ascribed to melano‐macrophages in fish (Bjørgen and Koppang 2024). On the other hand, melanin itself is powerful antioxidant, and the compound also has a great ability to act as an antioxidant and to bind chemicals including toxic compounds. If we see both these traits together, it is an appealing theory that newly formed melanin is important to protect the cell against self‐oxidative processes (Różanowska et al. 1999; Sarna and Swartz 2006), and to take this further, this can be especially important in fish macrophages. Fish contain high amounts of polyunsaturated fatty acids in their cell membranes, and in fish cells designed for phagocytosis with a machinery for generating acidic phagosomes and lysosomes, anti‐oxidative mechanisms may be even more important than in other cells. We have previously suggested that melanin may serve this purpose in fish (Bjørgen and Koppang 2024).

Thus, it should be evident that with the presence of melanin in inflammatory changes, it is important for the understanding of the condition to determine whether melanin is produced on site or is produced elsewhere and simply phagocytosed by macrophages. Naturally, this question also applies to a better appreciation of the mechanisms in melano‐macrophage centres (Agius and Roberts 2003; Steinel and Bolnick 2017). We recently revealed that melanin is indeed produced by the identical cells that also express the transcripts of the tyrosinase gene family in MFCs in salmon Bjørgen, Brimsholm, Asserson, et al. (2024). This observation points to a radical difference between inflammatory responses in fish versus mammals with respect to the involvement of the pigmentary system, as extracutaneous inflammatory reactions occur without melanisation in homeothermic vertebrates. However, the rationale for melanin presence in inflammatory changes in fish can so far only be speculated on but could be in line with the need for protection against oxidative changes. It has also been suggested that melanin could be regarded as a protective chemical filter which may explain its presence in sensitive tissues including the mammalian inner ear and in the *substantia nigra* in the central nervous system (Land, Ramsden, and Riley 2006).

## 4 Different Forms of Muscle Melanisation

**FIGURE 2:**
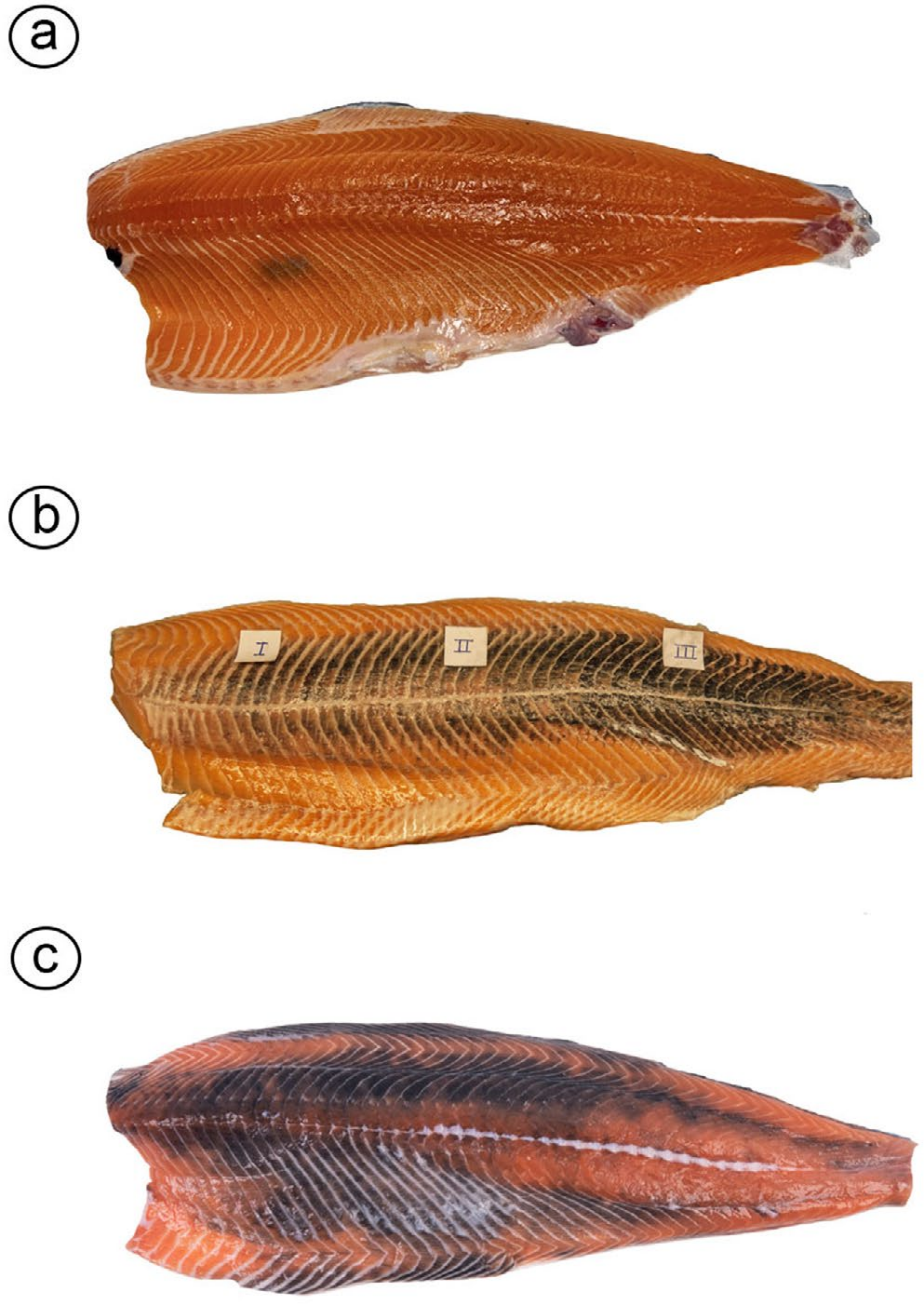
The different types of melanisation of the fillet. (a) A melanised focal change in the cranio‐ventral region of the white muscle. (b) Diffuse melanisation of the superficial red muscle. The white muscle is unaffected (image first published in Brimsholm, Rønning, et al. 2023). (c) Diffuse melanisation affecting major parts of the white muscle. Image kindly provided by Dr. Marcos Godoy, San Sebastián University, Chile.

Reports on chronic inflammatory changes with melanisation in smooth muscle are scarce in fish. To our knowledge, the involvement of intestinal smooth musculature and melano‐macrophages in conditions such as feed‐induced chronic inflammatory changes has not been documented (Bjørgen, Li, et al. 2020). As for cardiac muscle, inflammation induced by virus infections with the participation of melano‐macrophages has been noted (Fagerland et al. 2013). Nonetheless, most muscle changes associated with the presence of melano‐macrophages are observed in skeletal musculature, particularly in white skeletal muscle (Figure 2a).

Prior to the first report on MFCs (Koppang et al. 2005), a condition termed ‘black spot disease’ had been described in association with muscle reactions to encystation of metacercariae of digeneans (Turnbull 2006), in addition to melanised reactions towards foreign bodies (Roberts et al. 1973). In these cases, melanised cells appeared to attempt to encapsulate and isolate the foreign material from the surrounding tissue. Recently, a case report addressing muscle inflammation with melanisation due to intramuscular injection of vaccine was published (Mo et al. 2023), which is in line with previous reports on melanisation reactions towards the foreign material.

Among fish species, most studies on melanisation have concerned the Atlantic salmon, even though sporadic cases have also been observed in other species, for instance, in the southern sand flathead ( *Platycephalus bassensis* ) (Stocker et al. 2020), in the Atlantic cod ( *Gadus morhua* ) (Cooper and Midling 2007) and in farmed barramundi ( *Lates calcarifer* ) (Glencross et al. 2025). In Norway, a diffuse form affecting solely the red musculature has been reported (Brimsholm, Rønning, et al. 2023), but this manifestation seems to occur sporadically with low economic implications for the producers (Figure 2b). Also, sporadic cases of melanisation appearing along the spinal column and in the dorsal part of the fillet have been reported, but the most prevalent form is the MFCs affecting the cranio‐ventral and mid‐ventral part of the fillet (Mørkøre et al. 2015). This phenotype may also be encountered in the rainbow trout ( *Oncorhynchus mykiss* ), but in this species, the problem is negligible and has thus not been of any concern to the industry (Bjørgen, Brimsholm, Lund, et al. 2024). Consequently, research on muscle melanisation has primarily focused on the most prevalent form in Atlantic salmon, that is, MFCs, as evidenced by the literature referenced in the following sections. A diffuse form of melanisation affecting almost the entire fillet has recently caught attention in Chile and is considered an increasing problem with serious implications on fish welfare (industry representatives in Chile, personal communications to the authors) (Figure 2c).

## 5 What Are Red and Melanised Focal Changes?

RFCs and MFCs are discoloured lesions, either predominantly red or black, appearing in the white muscle. They have been reported in both farmed Atlantic salmon and farmed rainbow trout (Bjørgen, Brimsholm, Asserson, et al. 2024; Bjørgen, Brimsholm, Lund, et al. 2024; Koppang et al. 2005). They are most prevalent in the abdominal region in both species. In Mørkøre et al. (2015), it was reported that approximately 70% of the recorded MFCs in farmed salmon appeared in the anterior part of the fillet, with approximately 20% detected in the posterior abdominal musculature and the remainder in the dorsal musculature. No changes were reported in the caudal musculature. Bjørgen et al. (2019) found that 92% of the changes confined to the cranio‐ventral and mid‐ventral regions of the fillet. Changes affecting the peritoneum and underlying ribs are not included in this definition as the they are removed during processing at the abattoir. Only musculature changes beneath these structures are of significance to the industry and are registered as either RFCs or MFCs.

Under commercial and nonexperimental conditions, the first indication of the overall prevalence was provided by Koppang et al. (2005), who surveyed a commercial fillet production line for a period of 2 days and found that approximately 20% of the fillets were affected. Here, the authors also noted that the changes typically affected an area involving 2–5 myosepta. A national Norwegian registration surveillance from 2011 to and including 2015 showed variation along the costal line with highest prevalence in the south and an average national incidence increasing year by year from 13% in 2011 to 19% in 2015 (Mørkøre et al. 2015). Bjørgen et al. (2019) investigated the occurrence in a commercial fish farm and found an increase from 4% at 110 g fish with an increasing prevalence adding up to 30% before slaughter, when the fish had an average weight of 4.9 kg. This study showed that the MFCs accumulated over time in the population.

A registration protocol grading the changes was established by Mørkøre et al. (2015). Typically, these changes vary from 1 to 3 cm in diameter, and they may thus encompass several myotomes. However, larger changes may also appear, though less frequent (Bjørgen et al. 2015; Mørkøre et al. 2016). It has also been common practice to grade the changes as Grade 1, faint discolouration; Grade 2, distinct but not severe discolouration; and finally Grade 3 as prominent and severe discolouration (Bjørgen et al. 2019, 2015; Koppang et al. 2005). Regardless of macroscopic gradings, their histological appearance may vary considerably, and nine different histological categories have been described (Bjørgen et al. 2019).

## 6 Characteristics of Red Focal Changes (RFCs)

When we first began working with MFCs, haemorrhages were not studied in this context, as they were not a concern for the industry. However, over time, it became apparent that the MFCs were preceded by haemorrhages or RFCs. RFCs were first reported by Bjørgen et al. (2015) in a material of slaughter‐sized fish collected from different salmon producers together with wild‐caught fish devoid of changes. The changes were dominated by an acute haemorrhagic necrotic myositis. Vacuoles, presumably of lipid nature, were observed both in peripheral blood and between myocytes in relation to adipocytes. Histological staining for hemosiderin revealed reaction in the periphery of these vacuoles, showing presence of iron and indicating degradation of erythrocytes. In a follow‐up study, RFCs were registered and collected throughout the seawater phase by Bjørgen et al. (2019). The prevalence in the population was stable at approximately 4% throughout the sampling period, revealing that most RFCs were of similar character, although different degrees of haemorrhage, necrosis and inflammatory cell infiltrates were described.

Several theories for the development and occurrence of RFCs have been tested, but a breakthrough in our understanding of this condition came in 2023, when we applied spatial transcriptomic investigations. Here, based on KEGG (Kyoto Encyclopedia of Genes and Genomes) analysis, it was revealed that the hypoxia‐induced pathway 1 (HIF‐1) was upregulated (Bjørgen et al. 2023). These results were subsequently pursued in immunohistochemical analysis of RFCs targeting key proteins of the pathway, namely, HIF‐1α and two of its regulator proteins (prolyl hydroxylases 1 and von Hippel–Lindau), which were all highly expressed (Bjørgen, Brimsholm, Asserson, et al. 2024). The observed local haemorrhage and hypoxic conditions align with the characteristics of early‐stage fat necrosis, a phenomenon documented in various conditions affecting adipose tissue in both humans and animals. This entails a specialised necrotic reaction primarily targeting adipocytes (Taboada et al. 2009; Majithia et al. 2023). Although previous investigations had not focused on the adipose tissue involved in RFCs, our attention was swiftly turned towards understanding the role of adipocytes in the pathogenesis. Several striking histological similarities were detected in RFCs coinciding with descriptions of fat necrosis reactions, including necrotic adipocytes, haemorrhage and a myospherulosis‐like reaction (mixture of lipids and erythrocytes), and the formation of pseudocystic spaces (Bjørgen, Brimsholm, Asserson, et al. 2024). These are all early‐stage features of fat necrosis detected in RFCs, as described in conditions caused by fat necrosis of humans and other animals.

## 7 The Transition From Red to Melanised Focal Changes (MFCs)

The transition from red to melanised focal changes was first suggested by Bjørgen et al. (2015). In this study, some changes were identified with characteristics of both RFCs and MFCs within the same area, identified macroscopically as RFCs with some macroscopically observable melanisation. Histologically, early inflammatory changes with occasional melano‐macrophages were present. Similar changes were described by Bjørgen et al. (2019). Supporting evidence for the transition was published by Bjørgen, Brimsholm, Asserson, et al. (2024) when morphological approaches were applied to RFCs and MFCs targeting genes of the tyrosinase gene family encoding proteins in the melanin‐synthesising pathway. In melanocytes, these genes are expressed prior to the observation of melanin, and investigations showed that early‐stage RFCs were negative for these transcripts. However, as the changes progressed from the acute haemorrhagic/myocyte necrotic form and became more organised with infiltrating leukocytes, cells positive for the tyrosinase family genes were revealed by in situ hybridisation (Figure 1). In more advanced stages with faint melanisation in dispersed cells, these were also shown positive for the tyrosinase family genes. This finding indicated that cells first migrated to the site as an amelanotic phenotype following an establishment on site with melanin production (Bjørgen, Brimsholm, Asserson, et al. 2024). As such, these cells show a similar developmental pathway as cutaneous melanocytes which reach their destination as amelanotic cells before appearing melanised. Also here, in situ hybridisation for the tyrosinase family genes has been applied to provide evidence for this developmental sequence (Mort, Jackson, and Patton 2015; Steel, Davidson, and Jackson 1992).

In Bjørgen, Brimsholm, Asserson, et al. (2024), we also showed the presence of vacuoles or pseudocysts in RFCs. These are believed to occur due to necrosis of adipocytes, causing the release of lipase in the interstitium, which leads to triglyceride breakdown and the release of fatty acids accumulating in small pockets (Majithia et al. 2023). These pseudocysts, if not cleared by immune cells, can remain in the tissue and be further embodied in a fibrotic scar tissue and surrounded by pigmented cells in MFCs. Thus, the presence of pseudocysts in both RFCs and MFCs is yet another indication that these two presentations represent different stages of the same condition.

## 8 Characteristics of Melanised Focal Changes (MFCs)

**FIGURE 3:**
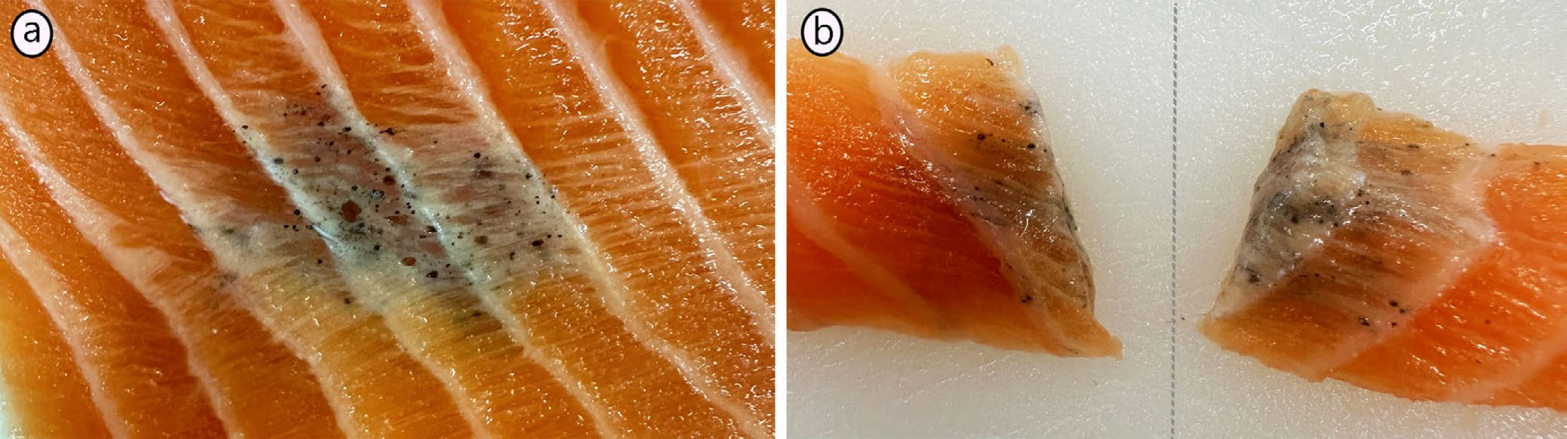
Typical gross appearance of a melanised focal change. (a) Discoloured change in the cranio‐ventral part of the fillet, featuring multiple spherical black structures located within the myosepta and extending into the myotomes. (b) The demarcated line indicates that the lesion has been sectioned in the middle of a myosepta, revealing the extent and depth of the melanotic changes. Images kindly provided by Ms. Charlotte Asserson, fish health biologist at Bremnes Seashore.

Before we understood the sequence of development from RFCs to MFCs, our focus was on MFCs alone (Figure 3). Consequently, the majority of published material centres around the characterisation of this form. From the initial investigation of the MFCs in 2005 (Koppang et al. 2005), it was evident that inflammation was a hallmark of the changes. These were characterised as chronic and granulomatous with presence of lipid vacuoles. Further, the changes were characterised by epithelioid cells and Langhan's giant cells and the presence of melanised cells containing melanosomes. In a later study, the MFCs were characterised as a chronic polyphasic necrotising myopathy (Larsen et al. 2012), and also here, large extracellular vacuoles containing lipids were described. The term ‘polyphasic’ indicates several different phases contained within the same lesion, that is, a large histological heterogeneity in the changes was evident. This histological heterogeneity was later addressed by Bjørgen et al. (2019), where a classification system was developed, comprising nine different categories with (1) no histological changes; (2) melano‐macrophages in the endomysium between apparently intact myocytes; (3) fibrosis in the endomysium without detection of melano‐macrophages; (4) fibrosis in the endomysium with melano‐macrophages; (5) melano‐macrophages, fibrosis, and presence of inflammatory cells in the endomysium; (6) (anticipated) old scar tissue with presence of inflammatory cells; (7) old scar tissue with presence of inflammatory cells and melano‐macrophages; (8) focal granulomatous inflammation with presence of melano‐macrophages; and (9) diffuse granulomatous inflammation with myocyte necrosis and myocyte regeneration and presence of melano‐macrophages. Notably, this study revealed that although MFCs might appear macroscopically similar and may obtain an identical macroscopic grading, the histological difference between such changes may be significant, ranging from the presence of occasional pigmented cells dispersed between seemingly unaffected myocytes to severe granulomatous inflammatory changes with abundant presence of melanised cells. These findings underscore the critical role of histopathological descriptions when investigating MFCs. With such a diversity, investigations of MFCs seem fruitless unless they are conducted within the framework of a histological characterisation.

Although the significance of fat necrosis in the development of MFCs was first suggested by Bjørgen, Brimsholm, Asserson, et al. (2024); Koppang et al. (2005) noted two different manifestations of vesicles in MFCs. One form was filled with macrophage‐like cells, debris and small droplets containing a homogenous material. Occasional haemorrhages were seen within these vesicles. The other form was round to oval and empty. Both forms were oil‐red O positive and surrounded with epithelioid‐like cells and fibro‐granulomatous tissue, melanised cells and occasional Langhan's giant cells. The authors believed that the lipid content was adjuvant oil from intraperitoneally administered vaccination; however, these two forms of lipid granuloma can also develop as a consequence of adipocyte necrosis. Here, the forms are known as typical and atypical lipogranuloma, the latter being hallmarked as cystic structures containing lipid and surrounded by epithelioid cells and giant cells. Giant cells of both foreign body type and the Langhan's type may occur (Horie 1954). As later studies showed that both vaccinated and unvaccinated fish may develop MFCs (Berg et al. 2012; Larsen et al. 2014), it seems plausible that the observed changes as described by Koppang et al. (2005) did not contain adjuvant oil, but rather fat from dead adipocytes. A number of studies have noted the lipid droplets in affected muscle (summarised in Table 1), but none of these have offered any further explanation for their existence before Bjørgen, Brimsholm, Asserson, et al. (2024) suggested them to result from a fat necrosis reaction. The early‐stage features of fat necrosis in RFCs were followed by the detection of late‐stage characteristics of fat necrosis in MFCs, including not only pseudocysts of various stages but also the presence of lipid‐laden macrophages (or foamy macrophages) engulfing the remnants of necrotic adipocytes. Although there is no experimental model available to study the progression of MFCs, the collective findings of key changes in a fat necrosis reaction as described in Bjørgen, Brimsholm, Asserson, et al. (2024) implicate its role as a driving factor in the development of the condition.

**TABLE 1:** Publications in peer‐reviewed journals addressing red and melanised focal changes in salmonids (in chronological order according to date of publishing).

Koppang et al. (2005) *Journal of Fish Diseases* 28: 13–22	First report of MFCs Macroscopic and histological description Identification of granulomatous inflammation with multinucleated giant cells, epithelioid cells, melano‐macrophages and oil droplets Detection of melanosomes Vaccination with oil‐adjuvanted vaccines assumed to be the cause of the condition
Berg et al. (2012) *Journal of Applied Ichthyology* 28: 446–452	Macroscopic observation of MFCs in groups of both unvaccinated and vaccinated fish with same frequency
Larsen et al. (2012) *Fish & Shellfish Immunology* 33: 277–285	Description of MFCs further classifying the inflammation with detection of transcripts for enzymes in the melanogenesis (eumelanin) pathway Presence of melanin was confirmed Granulomas with and without central lipid‐containing vacuoles were described
Larsen et al. (2013) *Developmental & Comparative Immunology* 41: 50–58	Melanogenesis in a melanin‐producing salmon macrophage cell line is influenced by temperature but not by virus infection or bacterin stimulation
Larsen et al. (2014) *Journal of Fish Diseases* 37: 327–340	Macroscopic observation and histological classification of MFCs in groups of unvaccinated and vaccinated fish showing no differences in either frequency or classification Smoltification at elevated temperatures identified as a risk factor Triploid fish more subjected to MFCs than diploid fish Large vacuoles interpreted as extracellular lipid was registered in moderate and severe changes
Bjørgen et al. (2015) *Veterinary Research* 46: 89	Bleedings in the muscle (RFCs) are identified to proceed the development of MFCs, so a sequence of development is established Piscine orthoreovirus 1 (PRV 1) is identified in the changes and concluded to be a premise for the development of severe MFCs Farmed fish in indoor tanks and wild fish are not found to have RFC or MFC The indoor tank fish is positive for PRV 1, so PRV 1 is not the initial cause of the condition
Krasnov et al. (2016) *Fish & Shellfish Immunology* 55: 56–63	Transcriptome profiling of MFCs showed inflammation, immune cell involvement with strong B‐cell responses Identification of prokaryotic rRNA in the changes Results indicated an initial trauma followed by a sustained immune response towards opportunistic microorganisms
Sissener et al. (2016) *Aquaculture* 464: 236–245	Fish fed a standard (8%) and low (5%) feed content of the *n* − 3 fatty acids EPA and DHA were compared at slaughter The prevalence of MFCs was increased in the low *n* − 3 diet There was no difference in the severity of the lesions (macroscopic observation) There was no difference in the prevalence of RFCs between the groups
Bjørgen et al. (2019) *Journal of Fish Diseases* 42: 935–945	In a study of a population over time, the frequency of RFCs is stable at about 4%, whereas that of MFCs increases over time PRV‐negative fish may develop both RFCs and MFCs, but the severity of the latter is aggravated with PRV MFCs may be histologically classified into nine different groups Injection of peripheral blood infected with PRV into the muscle does not induce the development of MFCs
Bjørgen, Kumar, et al. (2020) *Veterinary Immunology and Immunopathology* 222: 110035	With focus on RFCs, inflammatory cells including MHC class I and II+ cells, B cells and T cells were found to infiltrate the lesions at an early stage Genes related to innate immunity were upregulated at an early stage with a simultaneously strong downregulation of IL10 In MFCs, genes responsible for adaptive immune responses were upregulated PRV 1 was found to replicate centrally in melanised granuloma Large vacuoles and bleedings were observed in the RFCs without the involvement of inflammatory cells, indicating vert early stages of the condition Morphological investigations gave no indications for the involvement of bacteria
Malik et al. (2021) *Frontiers in Immunology* 12: 664624	RFCs contain abundant M1‐polarised macrophages, whereas MFCs contain a high number of M2‐polarised macrophages containing melanin There was a co‐localisation for PRV 1 with cells expressing CD8 and Mhc class I PRV 1 may induce a pro‐inflammatory environment that is important for the pathogenesis
Brimsholm, Rønning, et al. (2023) *Journal of Fish Diseases* 46: 453–458	A report on diffuse melanisation confined to red skeletal muscle Granulomas were not encountered; changes were dominated by fibrosis and scattered melano‐macrophages The detection of SAV2 and PRV1 suggested the involvement of these viruses in the pathogenesis
Lutfi et al. (2023) *British Journal of Nutrition*	The prevalence of MFCs was decreased in a fish group fed 3.5% EPA and DHA compared with a group fed 1.0%
Brimsholm, Fjelldal, Hansen, Trangerud, et al. (2023) *Anatomia Histologia Embryologia* 52: 421–436	A statistically significant association between costal fractures and both RFCs and MFCs was detected; however, such changes were also identified in fish with no fractures It was not determined if the costal fractures were primary or secondary to the soft tissue changes.
Jiménez‐Guerrero et al. (2022) *Aquaculture* 561: 738697	An association between rib abnormalities and MFCs was found, but additional factors influenced the changes
Hatlen et al. (2022) *Aquaculture* 560: 738555	The prevalence and physical size of MFCs was decreased in fish groups fed genetically modified canola oil
Brimsholm, Fjelldal, Hansen, Fraser, et al. (2023) *Journal of Fish Disease* 46: 1377–1389	Farmed (genetically selected), wild and hybrid salmon showed no differences in the incidence and severity of RFCs and MFCs raised under identical conditions A genetic component to the condition was thus ruled out Granulomas and vacuoles were observed in the material
Jiménez‐Guerrero et al. (2023) *Fish & Shellfish Immunology* 139: 108858	Several stages of B‐cell differentiation were identified in MFCs There was evidence for an active movement of B cells from MFCs to lymphatic organs and fat Bacteria were not detected in MFCs
Wakamatsu et al. (2023) *International Journal of Molecular Sciences* 24: 16797	Eumelanin was detected in MFCs but not in RFCs Some DOPA‐derived products detected in RFCs, suggestive of an oxidative environment
Bjørgen et al. (2023) *Cell and Tissue Research* 395: 199–210	RFCs and MFCs were analysed by spatial transcriptomics Hypoxia was targeted as a new feature of the changes
Bjørgen, Brimsholm, Lund, et al. (2024) *Diseases of Aquatic Organisms* 158: 201–213	When farmed under similar conditions, the prevalence of RFCs and MFCs is negligible in rainbow trout compared with the Atlantic salmon
Bjørgen, Brimsholm, Asserson, et al. (2024) *Journal of Fish Disease* 29: e13988	Histological evidence for hypoxia in the RFCs Different stages of fat necrosis identified in RFCs and MFCs Fat accumulations related to inflammatory changes Expression of tyrosinase and Tyrp1 in amelanotic cells in RFCs progress into detection of melanised cells in MFCs Fat necrosis identified as the driving force in the inflammatory process

## 9 Fate of Melanised Focal Changes (MFCs)

Bjørgen et al. (2019) showed that melanised focal changes accumulated in the fish over a time period of 1.5 years. During seven samplings throughout the seawater phase, the prevalence showed a steady increase. In macroscopically low‐graded changes (Grade 1), it could be difficult to observe any melanised cells at all, and when present, they could be dispersed between myocytes with no observable pathological changes. Given an elimination of the inflammatory‐triggering antigen, these findings might reflect a healing stage. However, Grade 1 changes accumulated over time, suggesting that such manifestations might have reached a stagnant stage.

Abundant melano‐macrophages and inflammatory changes were observed in macroscopically low‐graded changes (Grades 1 and 2) prior to PRV infection. Following PRV infection, also high‐graded changes (Grade 3) were observed in the population (Bjørgen et al. 2019). With such large, chronic changes and with encapsulation of lipid droplets and proliferation of PRV 1 within the granuloma (Bjørgen, Kumar, et al. 2020), it seems unlikely that these tissue changes could have restored back to a nonaffected stage.

These findings give reasons to believe that as with any inflammatory response, the possibility for a restoration of tissue integrity is present with MFCs. However, if lipid droplets and virus cannot be irradicated, the changes will persist over time. At an individual basis, features of the immune system and antigen load may determine the outcome.

## 10 Inflammatory Cells and Immune Reactions in Red and Melanised Focal Changes

RFCs are initiated by hypoxia and fat necrosis that my lead to myocyte degeneration and necrosis, and the changes are, therefore, not inflammatory driven from the start (Bjørgen, Brimsholm, Asserson, et al. 2024). However, following tissue injury, inflammatory cells are recruited to the lesions, seeking to restore tissue integrity, and the most significant finding in a study addressing immunopathological features of RFCs and MFCs was the absence of the cytokine IL10 expression in RFCs (Bjørgen, Kumar, et al. 2020). As IL10 downregulates immune responses, this reaction was interpreted as a key feature for the initiation of immune reactions in early stages. Most notably, the RFCs seem first to be infiltrated with Mhc class I and II positive cells believed to be macrophages, but also T and B cells have been noted in these changes, though with great variation between individuals (Bjørgen, Kumar, et al. 2020). Further, Bjørgen, Brimsholm, Asserson, et al. (2024) showed that cells expressing transcripts of the tyrosinase family genes but without any detectable melanin were present in early inflammatory infiltrates in the RFCs. This finding suggests that melanogenesis is a trait that develops very early in the condition.

As the RFCs progress into MFCs, we have more information on the immune responses as these changes have been subjected to investigations for a much longer time period. Importantly, cells with only sparce amounts of melanosomes expressing tyrosinase and Tyrp1 outdo a cellular component in the intermediate inflammatory response, whereupon heavily melanised cells can be detected at late stages of the inflammation (Bjørgen, Brimsholm, Asserson, et al. 2024). Simultaneously, the polarisation status of macrophages changes from M1 to M2 macrophages, the latter containing melanin (Malik et al. 2021). These cells are probably the same as identified in Bjørgen, Brimsholm, Asserson, et al. (2024) expressing tyrosinase and Tyrp1, but this has so far not been established in a joint investigation. Koppang et al. (2005) first investigated advanced MFCs by histological approaches. Here, a granulomatous inflammation dominated by epithelioid cells, Langhan's giant cells and the presence of melano‐macrophages were described. In MFCs, Mhc class II expression is higher than in RFCs, and the trend towards a chronic versus an acute response is evident in the expression of a number of different cytokines (Bjørgen, Kumar, et al. 2020).

Both T and B cells are important components of the inflammatory response in MFCs. This was established by Larsen et al. (2013), where transcription investigations and immunohistochemistry approaches for both cell types in addition to detection of MHC class II^+^ cells were applied. Similar investigations and findings in addition to investigations for some cytokines were reported by Larsen et al. (2014), and in 2016, Krasnov et al. (2016) confirmed these findings by demonstrating strong B‐cell responses in their material. Also, immune responses conformal with mild chronic inflammation initiated with trauma and bacterial or viral infection followed by immune responses to opportunistic microorganisms including Mhc class II activation were demonstrated. Bjørgen, Kumar, et al. (2020) showed abundant amounts of B cells in association with vacuoles (lipid droplets) in the changes. In 2023, several stages of B‐cell differentiation were detected in MFCs (Jiménez‐Guerrero et al. 2023); however, no histological investigations accompanied these results, and it is, therefore, not possible to relate them to any histological classification.

## 11 Factors Affecting the Pathogenesis of Melanised Focal Changes

Given that the search for aetiological causes and the underlying pathogenesis of MFCs has spanned over 20 years and has explored a wide spectrum of potential origins, we present the factors affecting the pathogenesis individually rather than in the chronological order that the research was conducted. The chronological sequence of published works is outlined in Tables 1 and 2. Following the summary of the knowledge regarding aetiological causes and factor affecting the pathogenesis, we conclude by presenting our current understanding of the development of these changes.

**TABLE 2:** Project reports and PhD dissertations addressing red and melanised focal changes.

Mørkøre et al. (2015) Mørke flekker i laksefilet. Kunnskapsstatus og tiltak for å begrense omfanget. Rapport 34/2015	MFCs occur early in the seawater phase and the prevalence increases towards time of slaughter MFCs occur in together with inflammatory changes and scar formation There are no significant effects of vaccine status or genetic background The development of MFCs can be affected by farming conditions, water quality, vaccination method health status and injuries
Mørkøre et al. (2016) Effekt av. fôr på melaninflekker i laks infisert med både PRV og SAV. Rapport 31/2016	Fish fed a high‐protein and low‐fat diet had significantly fewer and less severe MFCs compared with fish fed a standard diet Compared with standard diet, decreasing the fat level and increasing the protein level is advantageous with respect to frequency and severity of MFCs and also mortality and growth in PRV 1‐ and SAV3‐infected fish
Bjørgen et al. (2018) Faglig sluttrapport, FHF prosjektnummer 901221	RFCs and MFCs can be present in the fish regardless of PRV 1 infection The MFCs present themselves with different histopathological severity The most severe changes are always positive for PRV 1 Lipid vacuoles were registered in both RFCs and MFCs and assumed to play a role in the pathogenesis
Lund et al. (2018) Rapport 22018, Veterinærinstituttet, Oslo, Norway. FHF Project number 901256	The presence of MFCs in filet is multifactorial The risk factors vary between different data sets The prevalence is influenced by factors affecting the immune response, either directly at virus infections (the prevalence increases) or indirectly by the use of systemic health diets There is a positive effect of health diets
Mørkøre et al. (2022) EX‐spot: Mørke flekker i laksefilet. Årsak til dannelse og tiltak som hemmer utvikling. Rapport 27/2022	MFCs occur after transfer to sea, coinciding with increased rib fractures/abnormalities Lesions are characterised by accumulation of fat and connective tissue with altered structure, inflammatory cells and eumelanin Muscle bleedings caused by external trauma may develop into MFCs over time Leaner feed with high protein content reduces both the severity and prevalence of MFCs
*PhD dissertations*	
Hilde Anette Søiland Fagerland (2013)	Studies of extracutaneous pathological pigmentation—black spots—in Atlantic salmon. Dissertation for the degree of *Philosophiae Doctor* (PhD). Norwegian School of Veterinary Science, Oslo, Norway, 2013; ISSN 1890‐0364, ISBN 978‐7725‐253‐7
Håvard Bjørgen (2021)	Melanised focal muscle changes in Atlantic salmon—Interactions between infection and immunity. *Philosophiae Doctor* (PhD) Thesis. Norwegian University of Life Sciences, Faculty of Veterinary Medicine, Ås, Norway, Thesis number 2021:82; ISSN 1894‐6402, ISBN 978‐575‐1859‐2
Raoúl Jiménez‐Guerrero (2023)	Focal dark spots in the skeletal muscle of Atlantic salmon—involvement of rib abnormalities, environmental factors and local immune responses. *Philosophiae Doctor* (PhD) Thesis. Norwegian University of Life Sciences, Faculty of Biosciences, Ås, Norway, Thesis number 2023:67; ISSN 1894‐6402, ISBN 978‐82‐575‐2097‐7‐1859‐2
Malin Helen Brimsholm (2024)	Inflammatory conditions in the musculoskeletal system of salmonids in Norwegian aquaculture in relation to melanised changes. *Philosophiae Doctor* (PhD) Thesis. Norwegian University of Life Sciences, Faculty of Veterinary Medicine, Ås, Norway, Thesis number 2023:79; ISSN 1894‐6402, ISBN 978‐575‐2111‐0

### 11.1 External Factors

#### 11.1.1 Feed

The impact of feed ingredients on the occurrence and severity of MFCs was documented by Mørkøre et al. (2016). In their trials, a high‐protein and low‐fat diet had a significantly positive effect compared to a standard diet. The authors concluded that by increasing the protein level and decreasing the fat level in the feed, the condition improved. These findings were later repeated by Mørkøre et al. (2022). In this report, the authors stated that the standard feed protein and lipid combination was 35%/35%, whereas the high protein/low lipid feed combination was 45%/30% ‘in the period before slaughter’. The authors concluded that it seemed possible to reduce the prevalence of MFCs by half and to decrease their severity by providing the salmon with a leaner feed that had a higher protein concentration compared to the standard feed. These observations were made at a macroscopic level, and no histological evaluation was conducted. In two master's theses, the beneficial effect of feed antioxidants with respect to the occurrence of MFCs has also been noted (Rafiq 2015; Wang 2016).

Regarding the dietary content of the n‐3 long‐chain PUFAs EPA and DHA, Sissener et al. (2016) demonstrated a beneficial effect on the incidence of melanised focal changes with increased levels of these oils. There were no differences concerning the severity of the lesions, which were only assessed macroscopically. Interestingly, no differences in the prevalence of RFCs between the groups were observed. In accordance with these findings, Lutfi et al. (2023) reported a positive effect on the incidence of MFCs from feed containing elevated dietary levels of EPA and DHA in another material. Hatlen et al. (2022) investigated the effect of n‐3 canola oil in a 12‐month feeding experiment, showing positive effects both on the prevalence and severity of melanised focal changes with increasing oil levels added to the feed. The authors of these reports have discussed the possible explanations for the beneficial effects registered using these oils, suggesting that they might have a downregulation effect on the immune system. However, other yet unidentified factors could also play a role. None of these studies included histological investigations from the pathological changes, and classifications were based solely on naked‐eye observations. Histological investigations could have significantly elucidated the impact of the different diets on the pathogenesis and thus the cause of the condition.

The effect of feed was also noted in the surveillance study by Lund et al. (2018). Here, the use of ‘systemic health feed’ was observed to have a beneficial effect on the prevalence of MFCs. However, the authors emphasised that these results only indicated trends and studies would need to be continued for verification.

#### 11.1.2 Infectious Agents

Before it was known that MFCs developed from RFCs (Bjørgen et al. 2015), several attempts had been made to try to detect potential pathogens in the changes. In the initial report on the subject, Koppang et al. (2005) found no evidence for any infectious component. In a material collected by Larsen et al. (2012), RT‐qPCR for virus transcripts from salmonid alphavirus (SAV), infectious pancreatic virus necrosis virus (IPNV) and piscine orthoreovirus 1 (PRV 1) were negative. Different staining techniques aiming at identifying prokaryotic and eukaryotic micro‐organisms were also negative. However, Bjørgen et al. (2015) identified large amounts of PRV 1 in both RFCs and MFCs. Interestingly, melano‐macrophages were shown to be virus positive by immunohistochemistry. The authors concluded that the infection was a premise for the development of severe MFCs. This assumption was modified by Bjørgen et al. (2019) when following a fish population over time with sequential samplings. Initially, this population was PRV 1 negative but later became naturally infected with PRV 1. Both RFCs and MFCs occurred in the pre‐infection period. In the same report, it was also reported that injecting suspensions of PRV 1 into the musculature of salmon failed to produce MFCs. However, in the sequentially sampled population, the most severe changes (MFCs macroscopically Grade 3 and histologically Grade 7) only occurred after PRV 1 infection. In another follow‐up study, Bjørgen, Kumar, et al. (2020) detected replicating PRV 1 within Langhan's giant cells in well‐organised granuloma forming MFCs. This suggests that the immune system of the fish cannot eliminate the virus, but instead seem to wall it off and isolate it, in line with the development of granuloma. It is worth noting that rainbow trout, where the prevalence of MFCs is very low (Bjørgen, Brimsholm, Lund et al. 2024), does not get persistently infected with PRV 1 (Purcell et al. 2020).

The involvement bacteria has long been suspected in the development of MFCs, but early attempts searching for bacteria were negative. However, Krasnov et al. (2016) identified procaryotic rRNA in their material, leading these authors to suggest a bacterial component in the condition. In later studies, Bjørgen, Kumar, et al. (2020) and Jiménez‐Guerrero et al. (2023) investigated for the presence of bacteria and found none convincingly located in connection with the changes.

The National Veterinary Institute in Oslo conducted an epidemiological investigation and could not attribute MFCs to any specific cause. The risk factors varied between different datasets, but one common risk factor was attributed to virus infection. The prevalence of MFCs trended towards an increase when a fish population has experienced one, two or three virus infections, compared with fish where no viral disease had been diagnosed (Lund et al. 2018). However, no classifications of the MFCs were included in this study.

In summary, our current understanding regarding the involvement of infectious agents in the development of RFCs and MFCs suggests that PRV 1 infection should not be ignored as an important factor. Although the findings do not support that infection with this virus initiates the pathogenesis, PRV 1 seems to modulate the condition unfavourably, resulting in severe and unresolved MFCs.

#### 11.1.3 Vaccination

When first reported in 2005, the authors believed that the MFCs were due to vaccination (Koppang et al. 2005). The rationale for this assumption was, at this time, severe melanised inflammatory side‐effects that could be observed in fish having received oil‐adjuvanted intraperitoneal vaccination (Poppe and Breck 1997). Koppang et al. (2005) found the histological appearance of the changes to be very similar with fat vacuoles which was assumed to be lipids from vaccine oil adjuvant, and granulomatous inflammation with epithelioid cells and multinucleated Langhan's giant cells, indicative for antigen persistence. Micro‐organisms could not be detected, making the authors state that ‘we believe the pigmented changes encountered in salmon fillets are a consequence of vaccine localisation followed by a foreign body reaction’. However, 7 years later, Berg et al. (2012) reported that both vaccinated and nonvaccinated fish could develop MFCs. These observations were macroscopical, and it was thus unknown if these lesions were of the same nature as those described in the vaccinated fish previously. This question was resolved by Larsen et al. (2014) who investigated groups of vaccinated and unvaccinated fish. In this material, there was no significant difference in the incidence of MFCs between the groups. Also, the histological manifestations were undistinguishable. It was noted granulomatous inflammation with presence of melano‐macrophages in addition to ‘large extracellular vacuoles’ with melano‐macrophages surrounding them. The changes were thus clearly similar to those described by Koppang et al. (2005), mistaking them for containing adjuvant oil.

**FIGURE 4:**
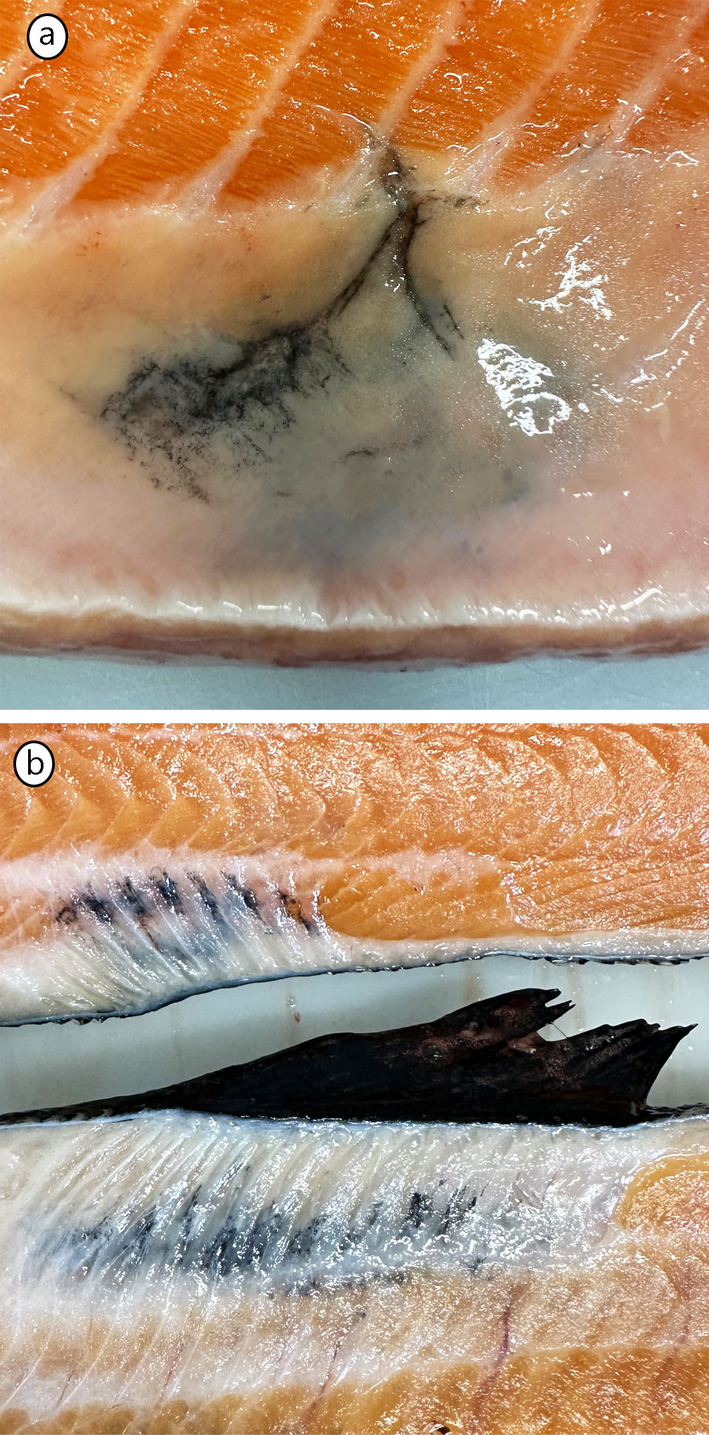
Melanised reactions post vaccination. (a) A melanised change appearing after intraperitoneal vaccination, erroneously affecting the peritoneal wall and underlying musculature. A discoloured linear lesion, presumed to be caused by the injection needle, is visible from the muscle extending towards the skin‐side (point of injection). (b) A melanised area confined to the connective tissue of the dorsal median septum surrounding the pterygiophores. Erroneous injection of oil‐based vaccine instead of DNA vaccine in this area can occur due to incorrect orientation of the fish during vaccination with multiple vaccines. Images kindly provided by Ms. Charlotte Asserson, fish health biologist at Bremnes Seashore.

While vaccination is not the primary cause of MFCs, it is well known that it can cause chronic inflammation and melanisation of the peritoneum, which also can impact the underlying musculature of the abdominal wall. Erroneous injection of the vaccine into the muscle may also occur, but in such cases, a characteristic melanised line in the muscle is typically seen, presumably marking the injection canal created by the needle and deposition of vaccine (Figure 4). Notably, Bjørgen et al. (2019) reported a peak in the prevalence of MFCs immediately after vaccination, suggesting that vaccination may influence the occurrence of MFCs at this stage of production. In a master's thesis, it was also noted a similar effect of vaccination (Jafelice 2014).

#### 11.1.4 Traumatic Injuries

Physical trauma has been repeatedly proposed as a probable cause of RFCs. Blunt trauma can occur in numerous ways, but notably, the involvement of ribs and bone fractures have been suggested as a potential source of haemorrhage. This could lead to a prolonged healing phase, resulting in MFCs. Both we and others have investigated for costal fractures associated with RFCs and MFCs, hypothesising that physical trauma and bone fracture may lead to the observed haemorrhages (Brimsholm, Fjelldal, Hansen, Trangerud, et al. 2023; Jiménez‐Guerrero et al. 2022). However, the results showed that RFCs and MFCs occurred both with and without costal fractures in the investigated material, questioning rib fractures as a primary cause of the condition. It is undeniable that ribs can be affected as part of the tissue response occurring in RFCs/MFCs, but this might be secondary to other aetiological factors. Again, it is worth noting that rainbow trout, where the prevalence of MFCs is very low (Bjørgen, Brimsholm, Lund, et al. 2024), lives under similar farming conditions as Atlantic salmon and would thus be just as exposed to mechanical trauma.

At Mowi, padding of exposed constructions in commercial steel‐cage net‐pens was implemented to assess whether trauma could be the causative factor. However, no effect was observed on the prevalence of MFCs in pens with padding versus normal commercial net‐pens (Ø. Oaland, Mowi ASA, personal communication).

#### 11.1.5 Environmental Factors

From the start of the recognition of the problem of MFCs, there has been a question regarding the occurrence of the condition in wild‐caught Atlantic salmon. It appears that the problem does not exist in the wild population. Bjørgen et al. (2015) found no changes in a limited number (*n* = 10) of wild‐caught Atlantic salmon from the River Drammenselven. More importantly, this study also referred to personal communication with Dr. E. Sterud from Norwegian Salmon Rivers, an association of Norwegian river owners, who reported no such findings in wild‐caught salmon. This situation remains the same to date (S. Hytterød, Norwegian Salmon Rivers, personal communication, June 2024). Together with the observation that genetically nondomesticated salmon in captivity may develop MFCs with a similar frequency as commercially bred fish (Brimsholm, Fjelldal, Hansen, Fraser, et al. 2023), we can conclude that the problem is related to farming conditions.

With respect to salmon kept in land‐based tanks, there are indications that there can be some beneficial effects on the condition. Changes were not present in 80 such fish individuals reared in small, indoor tanks (Bjørgen et al. 2015). Subsequent investigations have found that fish produced in small land‐based tanks (3–6 m^3^) with seawater may not develop any MFCs, whereas the prevalence is just as high as in fish in sea‐based commercial net‐pens in larger (1000 m^3^ and above) land‐based tanks. In small (125m^3^) sea‐based net‐pens, both the frequency and the severity of MFCs are reduced compared with larger net‐pens (1500 m^3^ and above) (Mørkøre et al. 2022). Thus, both on land and at sea, the condition seems to be aggravated in larger versus smaller production facilities. Industrial representatives have informed us of similar observations with respect to land‐based tanks and also that the prevalence is lowered in small, experimental net‐pens at sea (typically 5 + 5 m and 7 × 7 m). We can speculate that water current (indirectly causing increased exercise of musculature of the fish) and water oxygen saturation, which might be better in smaller facilities, may play a role in these observations.

### 11.2 Genetic Impact and Species Variation

Repeated speculations suggest that there could be a genetic component in the development of MFCs. Larsen et al. (2014) found that triploid fish were more prone to develop the condition than diploid fish. In a Nofima report from 2015, Mørkøre and co‐workers found no significant evidence for a genetic impact when examining different families of domesticated fish. As mentioned before, in wild‐caught Atlantic salmon, the problem appears to be nonexistent. As disease resistance is a selection criterion in domesticated fish, we hypothesised that such fish might be more susceptible to developing inflammatory conditions than wild fish. To investigate this, Brimsholm, Fjelldal, Hansen, Fraser, et al. (2023) conducted a study on genetically wild‐type salmon, domesticated salmon subjected to commercial selection programs and hybrid salmon produced from these two groups. These groups were then housed together in the same production facilities. The results showed no differences between the three groups regarding the prevalence of RFCs and MFCs, providing evidence to conclusively rule out a genetic component to this condition in Atlantic salmon.

Interestingly, rainbow trout farmed under the same conditions as Atlantic salmon, with similar feed and vaccine regimens, experiences minimal issues with MFCs. In rainbow trout, this condition is not recognised as a significant problem for the industry. To understand why this condition occurs in one salmonid species but not in another, we obtained samples from rainbow trout and registered the prevalence of such changes and classified them. The few pathological changes that we identified were similar in nature to those of salmon (Bjørgen, Brimsholm, Lund, et al. 2024). However, given the significant differences in lipid storing and metabolism between rainbow trout and Atlantic salmon, and the differences in susceptibility for certain viral diseases, we attributed this explanation as the cause for the observed prevalence differences between the two species (Bjørgen, Brimsholm, Lund, et al. 2024).

## 12 Muscle Inflammation or Pigmentation in Nonfish Production Animals

Concerns about fillet quality are not exclusive to farmed salmon production but also extend to terrestrial animals such as chicken. Despite their significantly shorter production time compared to farmed salmon, chickens can also develop substantial inflammatory changes in muscle, akin to changes seen in salmon. The condition in chicken is commonly known as white striping, a term that describes the macroscopic findings of white striations parallel to muscle fibres' direction (Kuttappan et al. 2013). Intriguingly, these changes have been reported more frequently in the cranio‐ventral region of the fillet. The incidence of these lesions increases with changes in meat composition, particularly a decrease in protein and an increase in fat percentages (Kuttappan et al. 2013).

Histologically, the changes share many features with MFCs in salmon. They are characterised by muscle degeneration, vacuolar degeneration (or pseudocysts), occasional mineralisation, regeneration of muscle fibres, mononuclear cell infiltration and fibrosis. Interestingly, the presence of fat, termed lipidosis in these publications, has been associated with increased severity of the lesions (Kuttappan et al. 2013). Although fat necrosis has not been proposed as the driving mechanism behind the development of these lesions, we assert that the same mechanism is at play here as in the MFCs of farmed salmon.

Muscle melanisation is a phenomenon not known in many other animals, which is to be expected, as the occurrence of melano‐macrophages is presumably restricted to fish, reptiles and amphibia (Bjørgen and Koppang 2024). But as a curiosity, melanised musculature is present in what is known as the black‐boned chicken. The phenomenon is associated with a genomic mutation, making melanocytes leaving the neural crest to follow an unusual migratory pattern and to also colonise extracutaneous tissues including muscle (Dorshorst et al. 2011). Thus, the musculature from these chickens appears black, which by tradition is considered to be a nutritionary beneficial trait in China (Deng et al. 2024) and which, unlike salmonids, is not a consequence of pathological changes in the tissue. The melanocytes present are normally dispersed between the myocytes. This hyperpigmentation results in aberrant immune responses (Shi et al. 2022), highlighting the crossover between the pigmentary and the immune system. In salmon, on the other hand, the presence of muscle melanisation is undoubtably a pathological condition.

## 13 Our Current Understanding of the Development of Melanised Focal Changes and the Road Ahead

**FIGURE 5:**
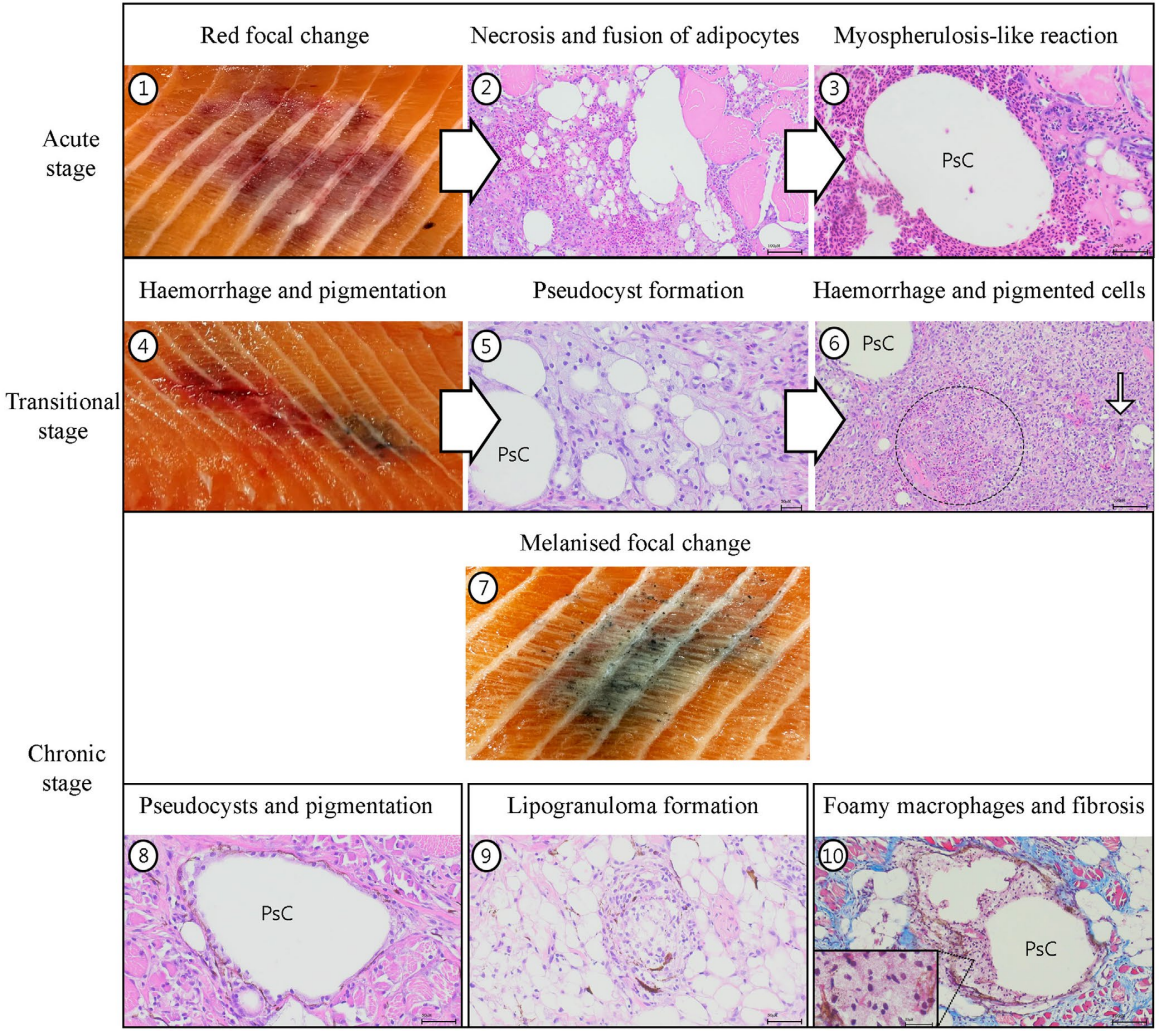
The proposed pathogenesis by Bjørgen, Brimsholm, Asserson et al. (2024), based on a fat necrosis reaction. The figure illustrates the initial acute phases in red focal changes (1–3), transitional stages (4–6) and chronic stages (7–9) of the condition. (1) Macroscopic focal haemorrhage affecting the myotomes and the myosepta. (2) Necrosis and fusion of adipocytes and initial pseudocyst formation. Scale bar—100 μm. (3) Myospherulosis‐like reaction with clustering of erythrocytes surrounding a lipid pseudocyst (PsC). Scattered inflammatory cells are present. Scale bar—50 μm. (4) Macroscopic transitional stage characterised by both haemorrhage and pigmentation. (5) Pseudocyst (PsC) formation is evident in addition to multiple small adipocytes and surrounding inflammatory cells. Scale bar—50 μm. (6) Haemorrhage is evident (dotted circle) with scattered melano‐macrophages (arrow) appearing in the tissue. Pseudocysts (PsC) are prevalent. Scale bar—50 μm. (7) Macroscopic focal pigmentation characterised by multiple spherical black structures appearing in both the myotomes and the myosepta. (8) Pseudocyst (PsC) surrounded by elongated melano‐macrophages and inflammatory cells. Scale bar—50 μm. (9) Organised lipogranuloma with macrophage‐like cells and melano‐macrophages appearing within the adipose tissue. Scale bar—50 μm. (10) Foamy macrophages in the process of clearing a pseudocyst within the adipose tissue. Fibrosis (blue colour) is elaborate. Scale bar—100 μm. High magnification image in lower left corner shows the presence of foamy macrophages with abundant finely vacuolated foamy cytoplasms and small, round centrally placed nuclei. Scale bar—25 μm.

For over two decades, substantial scientific efforts have been devoted to understanding the pathogenesis and the aetiology of the enigmatic MFCs occurring in farmed salmon. The recent discovery of fat necrosis marked a significant advancement (Bjørgen, Brimsholm, Asserson, et al. (2024), as it elucidates the pathological development underlying the condition (summarised in Figure 5). However, the initial causes for RFCs remain unresolved. Considering all the research that has been conducted and reviewed in this paper into account, we propose that the condition results from a combination of various production‐related factors that pave the way for a local fat necrosis reaction in the muscle. Environmental conditions and infections influence the development of these changes. Research into the interplay of these different factors will aid in ultimately resolving this problem. Future studies should include research into the feed lipid components in relation to the development of both RFCs and MFCs, and importantly be combined with meticulous histological examination. In addition, the direct relations between melanisation processes on one side and lipids and the immune system on the other side need to be explored to further our in‐depth understanding of the processes. Experiments in albino African clawed frog ( *Xenopus laevis* ) have pointed to the protective roles of melanin in lipid peroxidation processes (Corsaro et al. 1995), information that may be of high relevance in such studies.

## 14 Conclusion

Muscle melanisation in salmon presents a significant challenge for the economic profitability of the aquaculture industry and poses serious animal welfare issues. However, over the years, substantial progress has been made in understanding this condition. With recent findings highlighting the crucial role of a fat necrosis reaction within these changes, we now grasp the pathological mechanisms involved. Leveraging this knowledge in future experiments will hopefully equip us to reduce, or in the best‐case scenario, eliminate MFCs in farmed Atlantic salmon.

## Author Contributions

**Håvard Bjørgen:** writing – original draft, writing – review and editing, funding acquisition, visualization, conceptualization, formal analysis, project administration. **Espen Rimstad:** writing – review and editing, project administration. **Erling Olaf Koppang:** writing – original draft, funding acquisition, conceptualization, visualization, writing – review and editing, formal analysis, project administration.

## Ethics Statement

An ethical declaration is not required for this paper as it does not include any new results based on experimental or field trials. The images used are obtained from fish harvested at commercial abattoir plants, that is, nonregulated procedures according to the National Legislation on Animal Research.

## Conflicts of Interest

The authors declare no conflicts of interest.

## Data Availability

The authors have nothing to report.
